# ErbB receptor signaling directly controls oligodendrocyte progenitor cell transformation and spontaneous remyelination after spinal cord injury

**DOI:** 10.1002/glia.23586

**Published:** 2019-01-13

**Authors:** Katalin Bartus, Emily R. Burnside, Jorge Galino, Nicholas D. James, David L. H. Bennett, Elizabeth J. Bradbury

**Affiliations:** ^1^ King's College London, Regeneration Group, The Wolfson Centre for Age‐Related Diseases Institute of Psychiatry, Psychology & Neuroscience (IoPPN) London United Kingdom; ^2^ Nuffield Department of Clinical Neurosciences West Wing John Radcliffe Hospital Oxford United Kingdom

**Keywords:** ErbB tyrosine kinase receptor, neuregulin‐1, oligodendrocyte progenitor cells, Schwann cells, spinal cord injury

## Abstract

We recently discovered a novel role for neuregulin‐1 (Nrg1) signaling in mediating spontaneous regenerative processes and functional repair after spinal cord injury (SCI). We revealed that Nrg1 is the molecular signal responsible for spontaneous functional remyelination of dorsal column axons by peripheral nervous system (PNS)‐like Schwann cells after SCI. Here, we investigate whether Nrg1/ErbB signaling controls the unusual transformation of centrally derived progenitor cells into these functional myelinating Schwann cells after SCI using a fate‐mapping/lineage tracing approach. Specific ablation of Nrg1‐ErbB receptors in central platelet‐derived growth factor receptor alpha (PDGFRα)‐derived lineage cells (using PDGFRαCreERT2/Tomato‐red reporter mice crossed with ErbB3fl/fl/ErbB4fl/fl mice) led to a dramatic reduction in P0‐positive remyelination in the dorsal columns following spinal contusion injury. Central myelination, assessed by Olig2 and proteolipid protein expression, was unchanged. Loss of ErbB signaling in PDGFRα lineage cells also significantly impacted the degree of spontaneous locomotor recovery after SCI, particularly in tests dependent on proprioception. These data have important implications, namely (a) cells from the PDGFRα‐expressing progenitor lineage (which are presumably oligodendrocyte progenitor cells, OPCs) can differentiate into remyelinating PNS‐like Schwann cells after traumatic SCI, (b) this process is controlled by ErbB tyrosine kinase signaling, and (c) this endogenous repair mechanism has significant consequences for functional recovery after SCI. Thus, ErbB tyrosine kinase receptor signaling directly controls the transformation of OPCs from the PDGFRα‐expressing lineage into PNS‐like functional remyelinating Schwann cells after SCI.

## 1 INTRODUCTION

The lack of adequate therapies for spinal cord injury (SCI) is a major unmet need in medical research (Dietz & Fouad, 2014; Ramer, Ramer, & Bradbury, 2014). There is capacity for the activation of several intrinsic repair mechanisms after SCI (Hagg & Oudega, 2006; James et al., 2011; Weidner & Tuszynski, 2002) despite the immensely complex nature of its pathophysiology and associated severe neurological deficits (Fitch & Silver, 2008; Hagg & Oudega, 2006), providing a platform for some degree of spontaneous functional recovery (Fawcett et al., 2007). It is crucial to better understand the cellular and molecular mechanisms underlying endogenous repair events, which are suboptimal, and determine how they contribute to restoration of function. This may provide routes to modify and exploit these processes directly and to design combinations of effective therapeutic interventions that target different components of SCI pathology.

One spontaneous repair response triggered after SCI, albeit suboptimally, includes remyelination of surviving spinal axons which partially restores function (Assinck, Duncan, Plemel, et al., 2017; Bartus et al., 2016; James et al., 2011; Papastefanaki & Matsas, 2015; Plemel et al., 2014). Both oligodendrocyte‐ and Schwann cell‐mediated remyelination take place following SCI (Bunge, Bunge, & Ris, 1961; Bunge, Puckett, Becerra, Marcillo, & Quencer, 1993; Guest, Hiester, & Bunge, 2005), where it has been suggested that while oligodendrogenesis is an ongoing process in chronic SCI (Hesp, Goldstein, Miranda, Kaspar, & McTigue, 2015), Schwann cells may migrate into the injured spinal cord from the peripheral nervous system (PNS) following breakdown of the glial limitans (Franklin & Blakemore, 1993; Guest et al., 2005). However, in addition to infiltrating peripheral Schwann cells that may participate in central remyelination (Nagoshi et al., 2011), intrinsic newly formed remyelinating Schwann cells that arise from oligodendrocyte progenitor cells (OPCs) may also contribute (Zawadzka et al., 2010). Surprisingly, it was recently reported that oligodendrocyte‐mediated remyelination does not significantly contribute to functional improvements after SCI, potentially due to its delayed onset after injury (Duncan et al., 2018). In contrast, the onset of peripheral‐type remyelination is rapid, emerging within the first two weeks after injury and continuing over several months, and coincides with spontaneous locomotor recovery (Duncan et al., 2018; James et al., 2011). Thus, Schwann cell‐mediated remyelination of spinal axons may indeed drive at least a portion of recovery after SCI, as suggested previously (Bartus et al., 2016). Strategies to prevent demyelination at early stages and/or to accelerate and enhance myelin repair at later stages in order to limit progressive axonal degeneration are an important part of the wide spectrum of promising therapies for SCI (Assinck, Duncan, Hilton, Plemel, & Tetzlaff, 2017; Papastefanaki & Matsas, 2015). The view that axonal remyelination is an important element of regeneration after SCI has motivated clinical trials involving transplantation of neural precursor cells and Schwann cells (Bastidas et al., 2017; Cummings et al., 2005; Keirstead et al., 2005; Priest, Manley, Denham, Wirth, & Lebkowski, 2015; Santamaria, Solano, Benavides, & Guest, 2018; Sharp & Keirstead, 2007). However, understanding the underlying mechanisms of spontaneous remyelination after SCI may lead to alternative strategies to enhance this repair component, bypassing the need for transplantation (Assinck, Duncan, Hilton, et al., 2017).

Neuregulin‐1 (Nrg1), which signals via ErbB tyrosine kinase receptors, exists as multiple isoforms with diverse roles in nervous system development and function, playing an essential role in Schwann cell development and function in the PNS (Birchmeier & Bennett, 2016; Falls, 2003; Newbern & Birchmeier, 2010). We recently identified Nrg1 to be essential for spontaneous myelin repair processes after traumatic SCI (Bartus et al., 2016). Using conditional Nrg1 mutant mice, we demonstrated that injury‐induced Schwann cell remyelination of central axons in the dorsal columns is dependent on the presence of Nrg1 and contributes significantly to spontaneous locomotor recovery and improved axonal conduction after SCI (Bartus et al., 2016). We also found indirect evidence suggesting that many remyelinating Schwann cells associated with dorsal column axons after SCI are derived from newly dividing endogenous cells in the core of the SCI (Bartus et al., 2016). First, removal of adjacent dorsal roots (the source of potential infiltrating Schwann cells) did not affect the extent of Schwann cell myelin in the dorsal columns after SCI, and second, we observed newly dividing cells in close apposition to Schwann cell myelin rings in the SCI epicenter (Bartus et al., 2016). These findings are consistent with the observations made in a model of central focal demyelinating lesions (Zawadzka et al., 2010) and spinal cord contusion injury (Assinck, Duncan, Plemel, et al., 2017), and are also in parallel with the concept of endogenous neural stem cell‐like responses after central nervous system (CNS) injury that counteract injury‐induced tissue loss (Gregoire, Goldenstein, Floriddia, Barnabe‐Heider, & Fernandes, 2015). Under normal conditions, myelinating oligodendrocytes are the chief myelinating cells in the CNS and are derived from OPCs, characterized by their expression of NG2 and the platelet‐derived growth factor receptor alpha (PDGFRα) (Kang, Fukaya, Yang, Rothstein, & Bergles, 2010; Nishiyama, Lin, Giese, Heldin, & Stallcup, 1996; Rivers et al., 2008; Young et al., 2013). Using PDGFRα promoter/enhancer‐based fate‐mapping approaches, where PDGFRα‐expressing lineage cells are tracked in vivo, several recent studies have demonstrated considerable generation of both new myelinating oligodendrocytes (Assinck, Duncan, Plemel, et al., 2017; Hesp et al., 2015) and centrally remyelinating Schwann cells (Assinck, Duncan, Plemel, et al., 2017; Zawadzka et al., 2010) from PDGFRα‐expressing progenitor cells after SCI. Why and how progenitor cells of central origin can transform into remyelinating Schwann cells after CNS demyelinating injuries remains unknown. However, our previous findings showing that global deletion of Nrg1 entirely prevents Schwann cell remyelination of dorsal column spinal axons indicates that the Nrg1 signaling cascade plays a major role in this biological phenomenon.

We now aim to directly determine the molecular mechanism that drives the transformation of central progenitor cells into peripheral‐like Schwann cells capable of remyelinating spinal axons after SCI. Here, we test the hypothesis that Nrg1, via ErbB tyrosine kinase receptor activation, is the molecular switch that drives PDGFRα‐expressing central progenitor cells in the spinal cord to become remyelinating Schwann cells after SCI. We assessed this by conditionally ablating ErbB tyrosine kinase receptors in CNS resident glial progenitor/stem cells that express PDGFRα, which is not expressed by typical peripheral Schwann cells. We used lineage tracing PDGFRα mouse lines (PDGFRα/NG2) in which PDGFRα cells are labeled with tdTomato (Assinck, Duncan, Plemel, et al., 2017; Kang et al., 2010; Rivers et al., 2008; Zawadzka et al., 2010) crossed with ErbB3/4 receptor conditional mutant mice where ErbB3/4 receptors are ablated after tamoxifen dosing (Brinkmann et al., 2008; Riethmacher et al., 1997). Crossing these mutant lines generated mice in which ErbB receptor signaling is exclusively prevented in cells from the CNS resident PDGFRα‐expressing progenitor cell lineage. With this genetic fate‐mapping approach, we reveal that activation of ErbB tyrosine kinase receptors on PDGFRα‐expressing central progenitor cells directly controls their transformation into PNS‐like myelinating Schwann cells after contusive SCI. These central progenitor cells are presumably OPCs and are hereafter referred to as OPCs. However, given the heterogeneity of the PDGFRα progeny, we cannot exclude the possibility that other PDGFRα‐expressing progenitor cells may also be involved (Marques et al., 2016). Moreover, we provide direct evidence that remyelinating Schwann cells that are produced de novo in the injured spinal cord comprise a major proportion of the overall remyelinating Schwann cell population, and importantly these remyelinating Schwann cells significantly influence several aspects of spontaneous locomotor recovery, particularly those that depend on adequate proprioceptive input.

## 2 MATERIALS AND METHODS

### 2.1 Animals

All animal work carried out conformed to U.K. Home Office legislation (Scientific Procedures Act 1986). PDGFRαCreER^T2^/Tomato‐red reporter mice (to genetically label central PDGFRα‐expressing progenitor cells and their progeny) (Zawadzka et al., 2010) were crossed with ErbB3^fl/fl^/ErbB4^fl/fl^ mice (Brinkmann et al., 2008; Riethmacher et al., 1997). Crossing these two mouse lines produced a transgenic mouse line (referred to as PDGFRαCreER^T2^: ErbB3^fl/fl^/ErbB4^fl/fl^: tdTomato mice) in which, following tamoxifen administration, Nrg1 signaling is prevented at the receptor level specifically in PDGFRα‐expressing central progenitor cells. The progeny of these cells was fluorescently labeled by tdTomato. The use of these mice allows direct testing of our hypothesis that central progenitor cell transformation into central remyelinating Schwann cells is under the direct control of Nrg1 signaling following SCI. Mutant mice lacking ErbB3/4 receptors in PDGFRα‐derived cells were generated by administering tamoxifen (Sigma; 0.25 mg/g body weight in corn oil) by oral gavage for five consecutive days to 10‐week‐old PDGFRαCreER^T2^/Tomato‐red reporter mice and PDGFRαCreER^T2^:ErbB3^fl/fl^/ErbB4^fl/fl^: tdTomato mice, respectively. PDGFRαCreER^T2^ mice/Tomato‐red reporter mice with intact ErbB3/4 receptors served as tamoxifen controls. Tamoxifen was administered 4 weeks prior to surgery, ensuring gene recombination at the time of contusive SCI (Fricker et al., 2013).

### 2.2 Spinal contusion injury

Mice were anaesthetized with isoflurane, their backs were shaved and cleansed, and core temperature was maintained close to 37 °C using a self‐regulating heated blanket. Single doses of 0.05 mg/kg buprenorphine and 5 mg/kg carprofen were administered subcutaneously at the time of induction and the morning after surgery. Animals underwent midthoracic laminectomy and received a moderate midline 50 kDyne spinal contusion injury through the intact dura at the spinal level T10/11 using an Infinite Horizon's impactor (Precision Systems Instrumentation, Fairfax, VA, USA). Overlying muscle and skin were sutured in layers, subcutaneous saline was administered, and animals were left to recover from anaesthesia in a 37 °C incubator. Saline and enrofloxacin (5 mg/kg) were given subcutaneously daily for 3 and 7 days, respectively, after injury. Bladders were manually expressed two to three times daily during the first 2 to 3 days after surgery and once to twice daily thereafter, as required, until the end of the study period.

### 2.3 Behavioral assessments

#### 2.3.1 Basso mouse scale

The Basso Mouse Scale (BMS) (Basso et al., 2006) was used to assess open field hind limb locomotor function (*N* = 9–10 per group). This involved placing the animal in a circular open field (diameter ~1 m) and assessing both hind limbs during locomotion (over a 4 min session). Scores were calculated according to the 10‐point (0–9) BMS scale. For further detailed assessments of locomotor recovery, mice were also assessed with the 12‐point (0–11) BMS subscore scale, which further delineates recovery of specific locomotor features that may not be apparent in the overall BMS score (such as quantifying improvements in the areas of stepping frequency, coordination, paw position, trunk stability, and tail position). Testing was performed on Days 2, 5, and 7 after injury and once weekly thereafter for 8 weeks, by two experimenters blinded to the treatment groups. Animals were then sacrificed for immunohistochemical analysis. All BMS behavioral data are presented as mean ± standard error of the mean (*SEM*) values and statistical significance was accepted with *p* < .05 using two‐way repeated measures analysis of variance (anova) with Bonferroni post hoc tests. Statistical tests were performed using GraphPad Prism 7.

#### 2.3.2 Inclined beam‐walking test

For further detailed assessments of fine locomotor control, animals were also assessed on the inclined beam walking test. Beam‐walking apparatus consisted of an inclined beam (100 cm) fixed to a black “goal box.” The horizontal inclined beam consisted of a flat surface that gradually narrowed (1.5 cm broadest; 0.5 cm narrowest) and a small ledge underneath on either side. Animals were trained for seven consecutive days before baseline readings were obtained on two consecutive days, averaging three runs per testing day. After SCI, animals were assessed once they had regained weight‐supported stepping, averaging three runs per testing day. Before each post‐injury testing day, all animals were retrained for two consecutive days before scores were recorded. Animals were videotaped on each testing day in order to obtain scores. Left and right hind limb scores were calculated based on number of weight‐supported steps taken on the beam as well as lower scores (detailed below) for steps taken on the small ledges. The beam was divided into quarters; one point was scored for a weight‐supported step on the beam in the first broadest division. This score was doubled, tripled, or quadrupled in the second, third, and fourth sections of the beam due to the increased difficulty of the tapered beam. In all sections, one point was scored for a step taken on the small ledges. Data (*N* = 9–10 per group) are presented as mean ± *SEM* values and statistical significance was accepted with *p* < .05 using two‐way repeated measures anova with Bonferroni post hoc tests. Statistical tests were performed using GraphPad Prism 7.

### 2.4 Anatomical assessments

#### 2.4.1 Tissue preparation and immunohistochemistry

Animals were deeply anaesthetized with sodium pentobarbital (Euthatal: 80 mg/kg, i.p) and transcardially perfused with PBS (containing 0.1% heparin) followed by 4% paraformaldehyde in 0.1 M phosphate buffer. Immediately after perfusion, lesion site tissue was dissected (approximately 10 mm with the lesion epicenter located centrally). Tissue was postfixed overnight at 4 °C, cryoprotected in 30% sucrose for 48–72 hrs, then embedded and frozen in optimal cutting temperature (OCT) compound before being cut into serial transverse (20 μm) sections. Sections were immunostained using the following primary antibodies: rabbit monoclonal anti‐PDGFRα to label cells derived from the PDGFRα lineage (1:800, Cell Signaling Technology, London, UK), chicken polyclonal anti‐protein zero (P0) to label Schwann cell‐associated myelin (1:500, Abcam, Cambridge, UK), chicken polyclonal anti‐proteolipid protein (PLP) to label oligodendrocyte‐associated myelin (1:500, Millipore, Watford, UK), and rabbit polyclonal anti‐Olig2 to identify oligodendrocytes (1:500, Millipore). Complementary secondary antibodies were Goat polyclonal anti‐chicken IgY Biotin (1:500, Abcam) used in conjunction with ExtrAvidin FITC conjugate (1:500, Sigma, Dorset, UK), and donkey anti‐rabbit Alexa 488 (1:1000, Invitrogen, Thermo Fisher Scientific, Hemel Hempstead, UK). Briefly, after blocking with 10% donkey serum in PBS containing 0.2% Triton X‐100 (PBST) for 1 hr at room temperature (RT), the sections were incubated in PBST containing primary antibodies overnight at RT. After four washes of 5 min PBS, sections were incubated in PBST containing complementary secondary antibodies for 3 hrs at RT. After four washes of 5 min in PBS, sections were coverslipped with Vectashield mounting medium (Vector Laboratories, Peterborough, UK). Images were acquired using Nikon A1R Si Confocal Imaging system on an Eclipse Ti‐E inverted microscope.

#### 2.4.2 Immunohistochemistry image analysis

Quantification of P0 in injured mouse spinal cords (*N* = 8–9) was carried out by measuring the immunopositive areas in the dorsal column (AxioVision LE software), which were then expressed as percent of total dorsal column area. Total dorsal column area was measured by taking the mean area of intact dorsal column, measured rostral and caudal to the lesion site. Images were acquired sequentially, using the same exposure parameters.

Quantification of percent recombined cells in intact mouse spinal cords (*N* = 5 per group) was carried out by acquiring ×60 oil images taken randomly throughout the white matter, including dorsal, lateral, and ventral regions (3–4 images taken and quantified per sample). Images were acquired using Nikon A1R Si Confocal Imaging system on an Eclipse Ti‐E inverted microscope. Recombination efficiency was determined by counting the total number of PDGFRα‐positive and tdTomato‐positive cells and expressing the percent of tdTomato‐positive cell recombination relative to the total number of PDGFRα‐positive cells. PDGFRα and/or tdTomato‐positive vascular structures were not included in the cell counts.

Quantification of Olig2 positive‐cells in the lesion epicenter as well as Olig2 and tdTomato co‐expressing cells throughout the rostrocaudal lesion axis (*N* = 7 per group) was carried out on single channel maximum‐intensity z‐projections. For quantification of Olig2 positive‐cells, using batch macros and maintaining identical processing steps across all images, background was subtracted (rolling ball radius 10 px) a threshold representative of Olig2 positive immunostaining was applied and the Analyze Particles function in ImageJ utilized on a binary image to select cells (size 20–50 μm, circularity index 0.5–1). All processing steps were validated against manual cell counting in random sections with less than 5% error. For quantification of Olig2 and tdTomato co‐expressing cells, a mask was generated using these parameters and applied to a thresholded tdTomato channel using the ImageJ Transparent‐zero function. Double positive cells were again quantified using Analyze Particles. All anatomical quantification was carried out by an experimenter blinded to the treatment group and data are expressed as mean ± *SEM* values, using two‐way anova with Bonferroni post hoc tests for not repeated assessment and unpaired *t*‐test analysis as required. Statistical tests were performed using GraphPad Prism 7.

## 3 RESULTS

### 3.1 ErbB receptor signaling directly drives transformation of OPCs into remyelinating Schwann cells in the spinal dorsal columns after spinal contusion injury

It remains unknown how and why glial progenitor cells of central origin can transform into remyelinating Schwann cells after demyelinating injuries (Assinck, Duncan, Plemel, et al., 2017; Zawadzka et al., 2010). Given our recent evidence that global deletion of Nrg1 prevents Schwann cell remyelination in the dorsal columns after traumatic SCI, and that many remyelinating Schwann cells appear to be newly formed intrinsically within the spinal cord after injury (Bartus et al., 2016), we hypothesized that Nrg1 is a key molecular signal that underlies the differentiation of OPCs.

**Figure 1:**
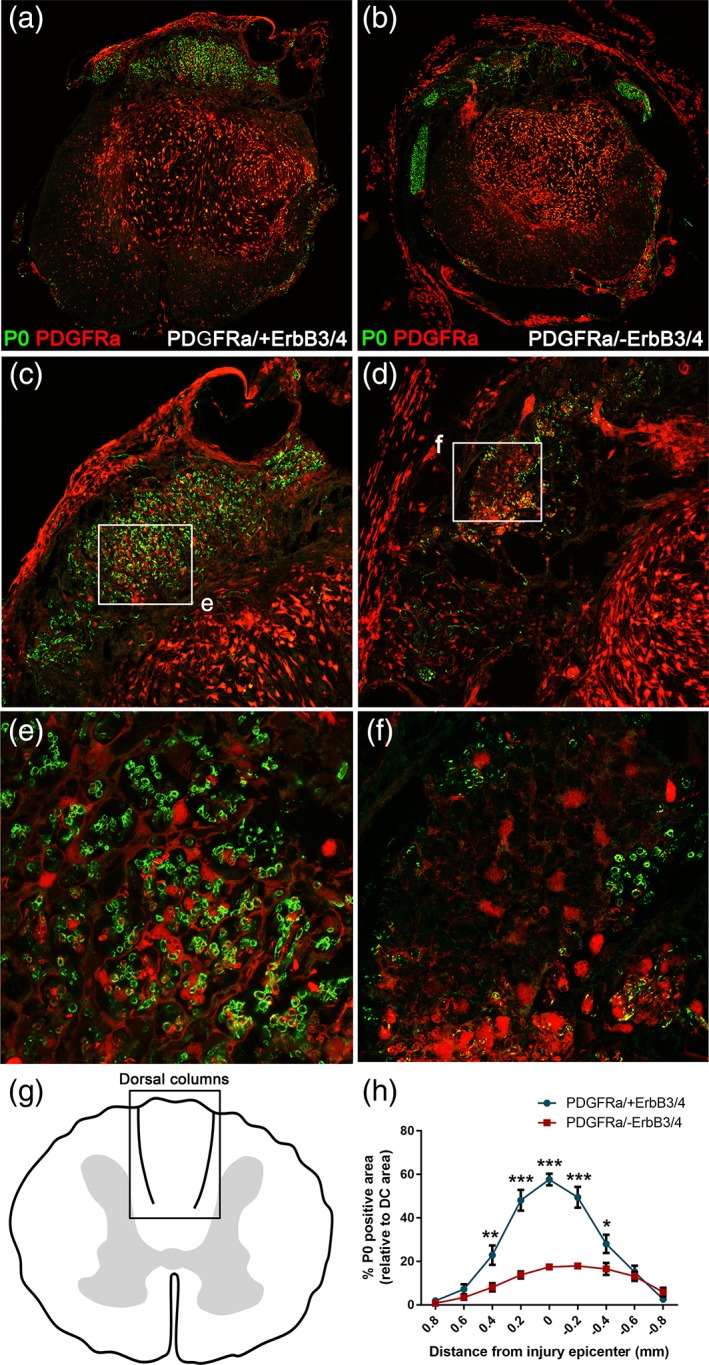
Neuregulin‐1 (Nrg1)‐ErbB receptor signaling directly controls the transformation of central progenitor cells into PNS‐like remyelinating Schwann cells in the dorsal columns after traumatic spinal cord injury (SCI). (a,c,e) Representative images from control (PDGFRα/+ErbB3/4) contused mouse spinal cords, showing typical Schwann cell‐associated myelin (P0, green) in the injured dorsal columns. (b,d,f) Representative images from injured mouse spinal cords in which ErbB receptors were lacking in PDGFRα‐expression central progenitor cells (PDGFRα/‐ErbB3/4). Specific ablation of ErbB receptors in central PDGFRα lineage progenitor cells (labeled with tdTomato, red) leads to dramatic reduction in remyelinating Schwann cells (P0, green) in the dorsal columns of contused mice. (g) Schematic indicating the dorsal column region where typical Schwann cell remyelination occurs and is quantified in order to evaluate the extent of Schwann cell‐mediated remyelination after injury. (h) Quantification of P0‐positive dorsal column area at the lesion epicenter reveals that most remyelinating P0‐associated Schwann cells are derived from central PDGFRα lineage cells, with only a minor population remaining following ablation of ErbB receptor in central progenitor cells and consequent inhibition of their transformation into remyelinating Schwann cells. Collectively, these data provide direct evidence that Nrg1‐ErbB receptor signaling controls the differentiation of centrally derived progenitor cells into peripheral‐like Schwann cells that remyelinate dorsal column axons after SCI

Previous studies that identified central progenitor‐derived Schwann cells during repair of focal CNS demyelination used genetic fate mapping of PDGFRα transgenic mice to identify CNS‐resident glial progenitor/stem cells (Zawadzka et al., 2010). Here, we crossed these transgenic mice in which the PDGFRα‐derived cells, upon tamoxifen administration, are genetically labeled with tdTomato (i.e., PDGFRαCreER line crossed with tdTomato reporter, termed PDGFRα/tdTomato mice) with mouse mutants lacking ErbB3/4 receptors (ErbB3^fl/fl^/ErbB4^fl/fl^ mice), to prevent Nrg1 signaling specifically in this population of PDGFRα‐expressing central progenitor lineage cells at the receptor level. Four weeks after 5 days of consecutive tamoxifen administration, all mice received a moderate 50 kDyn contusion injury at the spinal level T10. At 8 weeks post‐injury, specific ablation of ErbB receptors in PDGFRα‐expressing cells (labeled with tdTomato) led to a dramatic reduction in remyelinating Schwann cells (P0) in the dorsal columns of contused mice (Figure 1b,d) when compared with PDGFRα/tdTomato littermate controls that had intact ErbB3/4 receptors and the typical abundance of Schwann cell remyelinated axons (Figure 1a,c). High‐magnification images revealed a clear association of tdTomato‐positive cells with Schwann cell‐derived myelin within the dorsal columns in contused control mice (Figure 1e), which was significantly reduced in mice in which ErbB receptor signaling was inhibited specifically in PDGFRα lineage‐derived central progenitor cells (Figure 1f). However, some P0‐positive Schwann cell myelin not associated with tdTomato‐positive cells still remained in the dorsal columns of mice in which ErbB tyrosine kinase receptor signaling on PDGFRα‐derived central progenitor cells was blocked (Figure 1f), which are most likely a minor population of migratory preexisting Schwann cells that entered the injured spinal cord from the PNS (Assinck, Duncan, Plemel, et al., 2017; Nagoshi et al., 2011). Alternatively, given that not all cells were tdTomato‐recombined after tamoxifen treatment (Figure 3), the remaining Schwann cells may represent a mixed population of migrating preexisting Schwann cells and those that were newly formed from OPCs after injury where ErbB3/4 receptors remained intact. In total, approximately 58% of the dorsal column area quantified (Figure 1g) was occupied with P0‐positive Schwann cells in injured control mice at the lesion epicenter (signified by point zero), which was greatly reduced to approximately 17% in mice lacking ErbB tyrosine kinase receptors in PDGFRα‐expressing lineage cells (Figure 1h). The extent of Schwann cell remyelination was consistently and significantly reduced throughout the entire rostrocaudal axis of the spinal lesion (Figure 1h; two‐way anova with Bonferroni post hoc, *p* < .001, *N* = 8–9, PDGFRα/+ErbB3/4 versus PDGFRα/‐ErbB3/4). This suggests that the majority of remyelinating Schwann cells in the injured spinal cord are intrinsically produced from central OPCs after SCI.

### 3.2 ErbB receptor signaling does not significantly alter oligodendrocyte production at the injury site after spinal contusion

**Figure 2:**
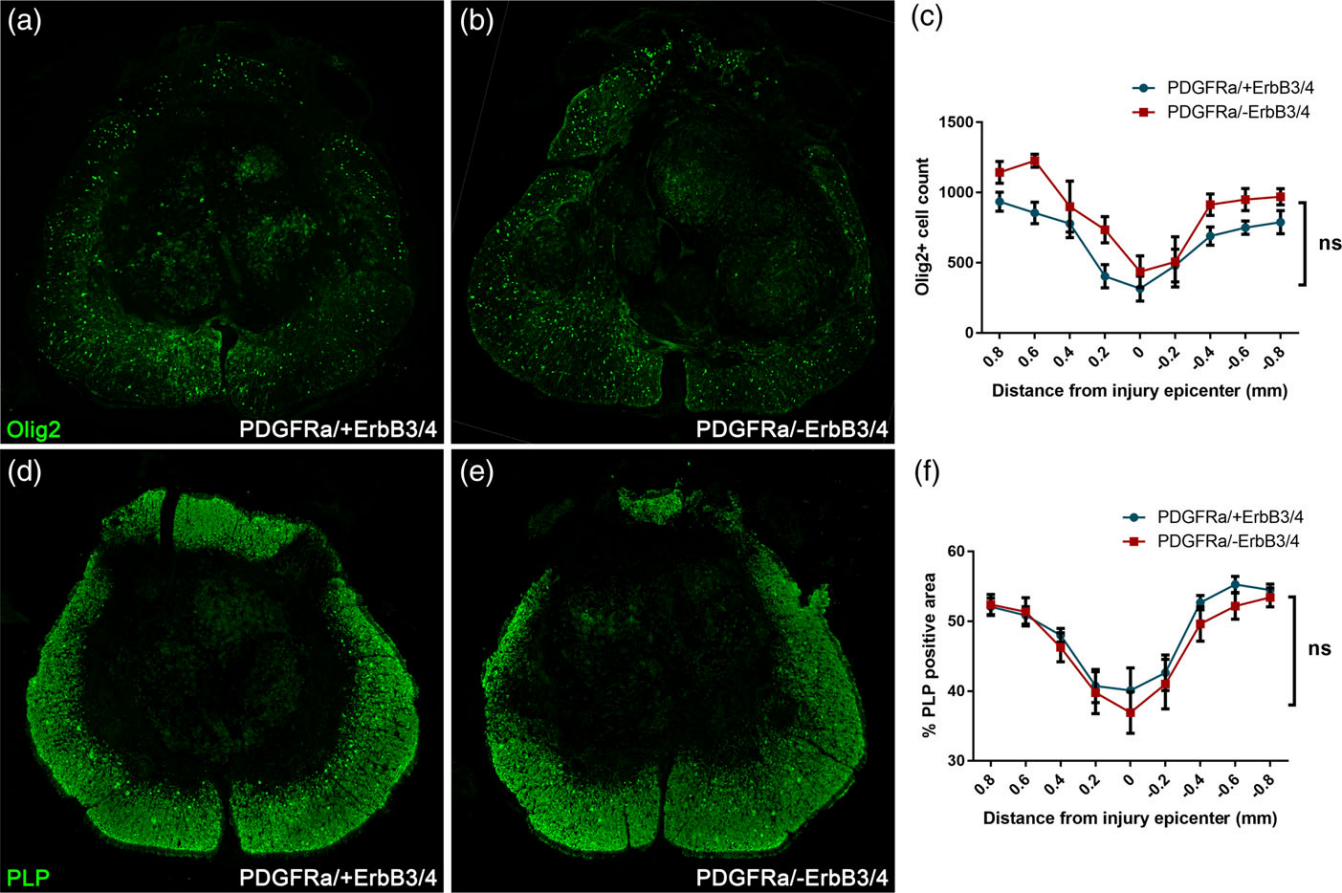
Selective removal of ErbB receptor signaling in central progenitor cells (PDGFRα/‐ErbB3/4) does not significantly alter oligodendrogenesis at the lesion epicenter where prominent Schwann cell remyelination is taking place. Representative images taken from the lesion epicentre from control injured mice (PDGFRα/+ErbB3/4; a) and injured mice lacking PDGFRα‐expression in central progenitor cells (PDGFRα/‐ErbB3/4; b), showing staining for oligodendrocytes (Olig2, green). (c) Quantification of Olig2‐positive cells suggests no significant difference in the number of oligodendrocytes at the lesion epicentre and throughout the rostrocaudal lesion axis. Representative images taken from the lesion epicentre from control injured mice (PDGFRα/+ErbB3/4; d) and injured mice lacking PDGFRα‐expression in central progenitor cells (PDGFRα/‐ErbB3/4; e), showing staining for central myelin (PLP, green). (f) Quantification of PLP‐positive area suggests no significant difference in oligodendrocyte central myelination at the lesion epicentre and throughout the rostrocaudal lesion axis

It is known that PDGFRα‐positive OPCs not only give rise to myelinating Schwann cells in the injured spinal cord (Assinck, Duncan, Plemel, et al., 2017; Zawadzka et al., 2010) but also produce de novo remyelinating oligodendrocytes at the lesion epicenter (Assinck, Duncan, Plemel, et al., 2017). Furthermore, recent evidence suggested that Nrg1 promotes oligodendrocyte‐mediated remyelination after SCI (Kataria et al., 2018). Therefore, we assessed whether ErbB receptor signaling also affects oligodendrocyte remyelination after SCI. Specific ablation of ErbB3/4 receptors in PDGFRα‐expressing progenitors did not significantly alter the number of Olig2 positive oligodendrocytes throughout the rostrocaudal axis of the spinal lesion in contused mice at 8 weeks post‐injury (Figure 2a–c; two‐way anova, *p* > .05, *N* = 7, PDGFRα/+ErbB3/4 versus PDGFRα/‐ErbB3/4). We also evaluated the extent of central myelination throughout the rostrocaudal axis of the injury by quantifying the amount of the oligodendrocyte myelin marker PLP at 8 weeks post‐injury and found no significant difference between groups (Figure 2d–f; two‐way anova, *p* > .05, *N* = 7, PDGFRα/+ErbB3/4 versus PDGFRα/‐ErbB3/4).

**Figure 3:**
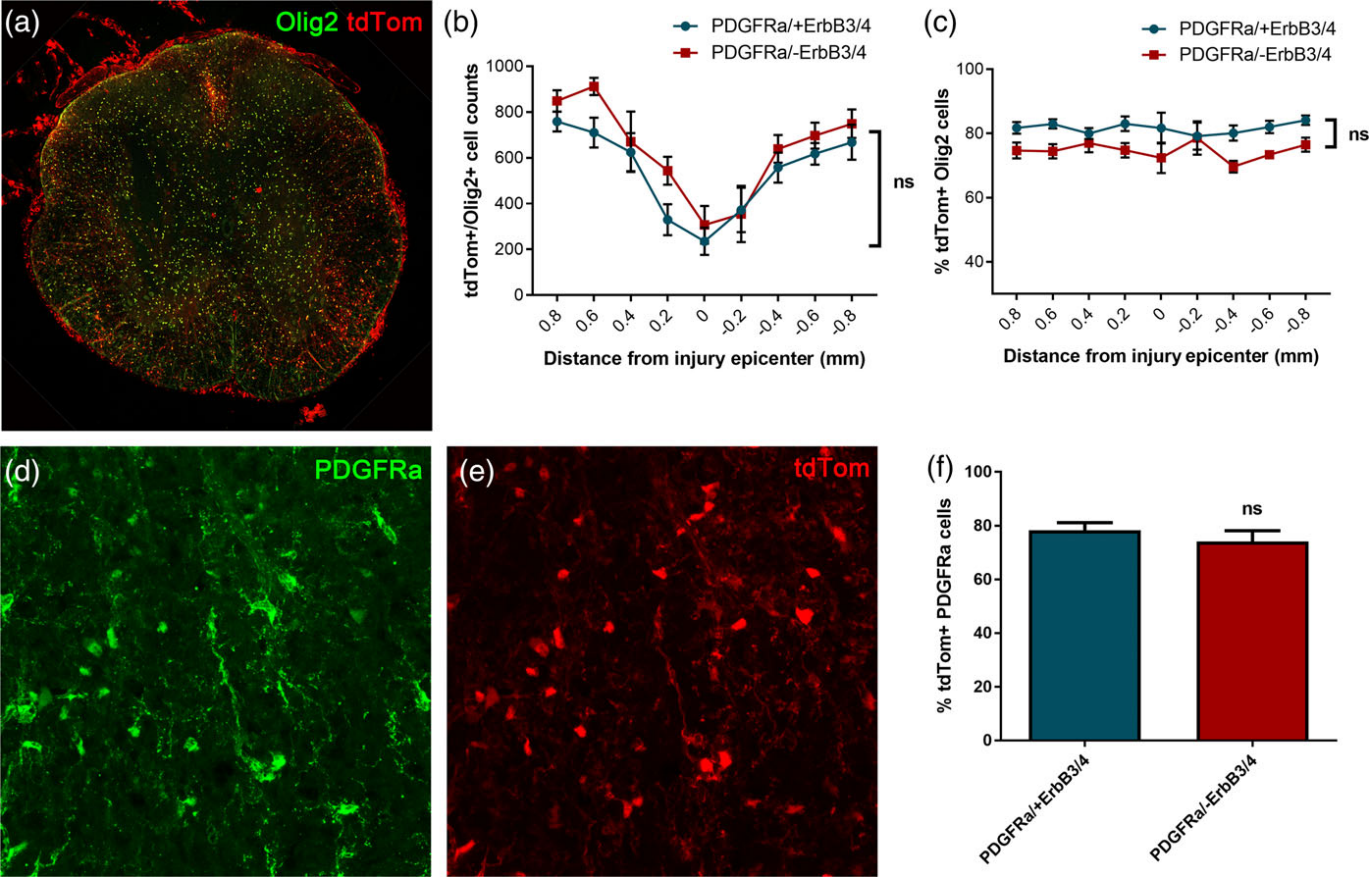
Recombination efficiency in PDGFRαCreER/tdTomato reporter mice crossed with ErbB3^fl/fl^/ErbB4^fl/fl^ mice. (a–c) Olig2‐ and tdTomato co‐expression throughout the rostrocaudal lesion axis in injured control mice (PDGFRα/+ErbB3/4) and mice lacking ErbB receptors in PDGFRα‐expressing central progenitor cells (PDGFRα/‐ErbB3/4), showing no significant differences. (d–f) Percent of PDGFRα‐expressing cells recombined as evaluated by tdTomato co‐expression in injured control mice (PDGFRα/+ErbB3/4) and mice lacking ErbB receptors in PDGFRα‐expressing central progenitor cells (PDGFRα/‐ErbB3/4)

To assess recombination efficiency, we determined the number and percentage of tdTomato‐positive (PDGFRα progeny) cells that also expressed the oligodendrocyte marker Olig2 throughout the rostrocaudal axis of the spinal lesion at 8 weeks post‐injury (Figure 3a–c), as well as the extent of tdTomato and PDGFRα double‐positivity in the uninjured spinal cord (Figure 3d–f), both in PDGFRα/tdTomato mice with intact ErbB3/4 receptors and those specifically lacking ErbB3/4 receptors in PDGFRα‐expressing progenitors. There were no significant differences between groups, finding approximately 75%–80% tdTomato‐positive cells that co‐expressed Olig2 throughout the rostrocaudal extent of the lesion axis (two‐way anova, *p* > .05, *N* = 7, PDGFRα/+ErbB3/4 versus PDGFRα/‐ErbB3/4; Figure 3c) and approximately 75%–80% PDGFRα‐positive cells that co‐expressed tdTomato in intact spinal cord (percent of tdTomato‐positive cell recombination 78.3 ± 2.9 and 74.1 ± 4.1 for PDGFRα/+ErbB3/4 and PDGFRα/‐ErbB3/4, respectively; unpaired *t* test, *p* > .05, *N* = 5 per group; Figure 3f). Comparable recombination efficiency in uninjured mouse spinal cord was reported in a previous study using the PDGFRα/tdTomato mice, who reported 84% recombination (Assinck, Duncan, Plemel, et al., 2017).

### 3.3 Ablation of Schwann cell‐dependent remyelination after spinal contusion injury impairs aspects of spontaneous locomotor recovery that depend on proprioceptive motor control

**Figure 4:**
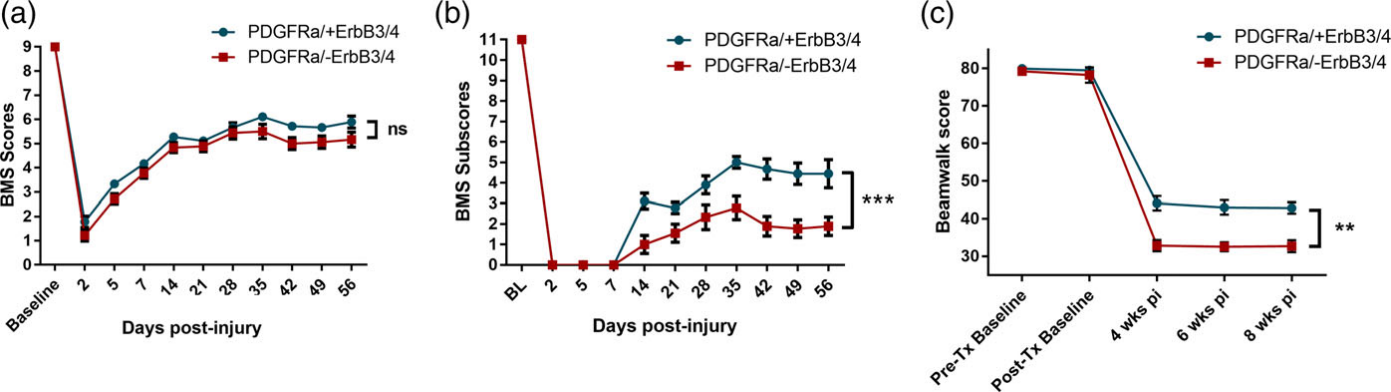
Selective removal of ErbB receptor signaling in central progenitor cells (PDGFRα/‐ErbB3/4) leads to significantly poorer functional outcome in spinal injured adult mice, especially that greatly rely on proprioceptive dorsal column function. (a) Gross BMS scores in injured control mice (PDGFRα/+ErbB3/4) and mice lacking ErbB receptors in PDGFRα‐expressing central progenitor cells (PDGFRα/‐ErbB3/4), showing no statistically different functional improvements between groups. (b) BMS subscores in injured control mice (PDGFRα/+ErbB3/4) and mice lacking ErbB receptors in PDGFRα‐expressing central progenitor cells (PDGFRα/‐ErbB3/4), revealing a greater deficit in mice lacking ErbB receptors in central progenitor cells. (c) Beam‐walking scores in injured control mice (PDGFRα/+ErbB3/4) and mice lacking ErbB receptors in PDGFRα‐expressing central progenitor cells (PDGFRα/‐ErbB3/4), showing significantly poorer performance of mice lacking ErbB receptors in central progenitor cells. Mice were tested pre‐injury before and after tamoxifen (Tx) dosing up to 8 weeks post‐injury (pi) in all behavioral assessments. Collectively, these data provide direct evidence that Nrg1‐ErbB signaling‐dependent endogenous repair by CNS progenitor cells in this spinal cord region has significant consequences for functional recovery that relies on proprioceptive dorsal column function

To assess whether centrally derived remyelinating Schwann cells could contribute to functional recovery after SCI, we monitored injured mice for spontaneous locomotor recovery using the inclined beam‐walking test as described previously (Bartus et al., 2016), the 10‐point BMS, and 12‐point BMS open field hind limb locomotion subscore, to delineate recovery of fine locomotor features that are not apparent in the overall BMS score (Bartus et al., 2016; Basso et al., 2006). Assessment of open field locomotion during 8 weeks of post‐injury recovery using the BMS scale revealed no significant differences in locomotor recovery between injured control animals and animals lacking ErbB receptors in PDGFRα‐expressing progenitor cells (Figure 4a; two‐way repeated measures anova, *p* > .05, *N* = 9–10, PDGFRα/+ErbB3/4 versus PDGFRα/‐ErbB3/4). BMS subscore analysis after contusive SCI, however, showed significantly worsened functional recovery in injured PDGFRα/‐ErbB3/4 animals compared with injured control animals (Figure 4b; two‐way repeated measures anova, with Bonferroni post hoc, *p* < .001, *N* = 9–10, PDGFRα/+ErbB3/4 versus PDGFRα/‐ErbB3/4; average score at 8 weeks post‐injury 4.4 ± 0.7 and 1.9 ± 0.5, respectively). Furthermore, assessment of functional performance on the beam walking task, which requires weight‐supported stepping ability, coordination, balance, and proprioception (locomotor features that rely on dorsal column function [Kanagal & Muir, 2008]) revealed reduced beam‐walking ability after SCI both in PDGFRα/tdTomato control mice with intact ErbB receptor signaling (PDGFRα/+ErbB3/4 mice), as well as in PDGFRα/tdTomato mice in which ErbB receptor signaling was prevented in central progenitor cells (PDGFRα/‐ErbB3/4 mice; Figure 4c). However, mice lacking ErbB3/4 receptors on OPCs (with decreased Schwann cell remyelination of dorsal column axons) were significantly more impaired at each time point tested than mice with intact ErbB3/4 receptors (Figure 4c; two‐way repeated measures anova, with Bonferroni post hoc, *p* < .001, *N* = 9–10, PDGFRα/+ErbB3/4 versus PDGFRα/‐ErbB3/4; average score at 8 weeks post‐injury 43 ± 1.5 and 33 ± 1.6, respectively, compared with post‐tamoxifen baseline scores of 80 ± 0.8 and 78 ± 2.1, respectively). These data demonstrate significantly worse spontaneous functional outcome in PDGFRα/‐ErbB3/4 mice (which lack Schwann cell remyelination in the dorsal columns) than in PDGFRα/+ErbB3/4 mice (with typical Schwann cell remyelination in the dorsal columns), particularly in tasks that require dorsal column function.

## 4 DISCUSSION

In light of our previous findings which showed that selective ablation of Nrg1 prevents spontaneous Schwann cell mediated remyelination after SCI (Bartus et al., 2016), our data now reveal that ErbB tyrosine kinase receptor activation controls the differentiation of centrally derived progenitor cells from the PDGFRα lineage, presumably OPCs, into PNS‐like Schwann cells that remyelinate CNS axons after traumatic SCI and significantly contribute to spontaneous locomotor recovery. These data reveal a novel role for ErbB tyrosine kinase receptor signaling after CNS injury, providing important mechanistic insight as to the molecular signaling driving spontaneous regenerative remyelination and functional repair after SCI which may also have important implications in other CNS disorders with a demyelinating pathology.

It is widely established that remyelination of demyelinated axons within the injury penumbra of the spinal cord is an important regenerative process, described in several animal models of SCI (Bunge et al., 1961; James et al., 2011; McDonald & Belegu, 2006; Papastefanaki & Matsas, 2015; Plemel et al., 2014). However, some axons reportedly remain chronically demyelinated, in several animal models (Blight & Decrescito, 1986; James et al., 2011; Nashmi & Fehlings, 2001; Waxman, 1989) and up to a decade after human SCI (Bunge et al., 1993; Guest et al., 2005). It is also known that myelin integrity is essential for efficient fine tuning of motor skills and sensory integration, and there is evidence that remyelination restores functional outcome (Blight & Young, 1989; Duncan, Brower, Kondo, Curlee, & Schultz, 2009; Honmou, Felts, Waxman, & Kocsis, 1996; Karimi‐Abdolrezaee, Eftekharpour, Wang, Morshead, & Fehlings, 2006; Keirstead et al., 2005; Smith, Blakemore, & McDonald, 1979) and is crucial for axonal protection and metabolic support (Franklin & Ffrench‐Constant, 2008; Irvine & Blakemore, 2008; Nave, 2010; Quintes, Goebbels, Saher, Schwab, & Nave, 2010). Following acute demyelination, there is a phase of remyelination that is not exclusively carried out by oligodendrocytes. Schwann cells, which are not usually present within the CNS, also remyelinate central spinal axons, observed in animal models and human postmortem samples following SCI (Bunge et al., 1993; Guest et al., 2005; James et al., 2011; McDonald & Belegu, 2006) and other demyelinating pathologies (Fehlings & Skaf, 1998; Ghatak, Hirano, Doron, & Zimmerman, 1973; Itoyama, Ohnishi, Tateishi, Kuroiwa, & Webster, 1985). Evidence demonstrates that central axons that have been remyelinated by Schwann cells have normal myelin properties (Blight & Young, 1989; Felts & Smith, 1992, 1996) and regain conduction (Bartus et al., 2016; Blight & Young, 1989; James et al., 2011).

We have recently identified Nrg1 to be a key molecule driving this Schwann cell‐mediated remyelination after SCI, and that this mediates the restoration of axonal conduction through the lesion site and the degree of spontaneous locomotor recovery (Bartus et al., 2016). At least some of these centrally myelinating Schwann cells may migrate into the CNS following breach of the glia limitans, although recent evidence using models of chemically induced primary demyelination suggest that they may also arise from progenitor cells within the CNS (Zawadzka et al., 2010). In the current study, we now demonstrate that this is mediated by ErbB tyrosine kinase receptor signaling on OPCs, and this regulates de novo production of a population of remyelinating Schwann cells within the injured spinal cord. Selective removal of ErbB3 and ErbB4 signaling in this CNS progenitor population not only prevented the majority of Schwann cell‐mediated remyelination in the injured dorsal columns but also had a significant functional impact on locomotor recovery after injury, particularly in tasks dependent on dorsal column function. These findings are comparable to our previously reported results in which the ligand Nrg1 was conditionally ablated (Bartus et al., 2016). However, while conditional global ablation of Nrg1 led to complete lack of Schwann cell remyelination after SCI (Bartus et al., 2016), specific ablation of ErbB receptors in PDGFRα‐expressing progenitor cells preserved some remyelinating Schwann cells in the injured spinal cord. This could be explained by the fact that axonal Nrg1 expression is an absolute necessity for Schwann cell‐axon attraction and myelination, regardless of the Schwann cell phenotype or origin (Ulanska‐Poutanen et al., 2018). We acknowledge, moreover, that although Nrg1 typically engages ErbB tyrosine kinase receptors, other ligands (e.g., Nrg2, 3, and 4 as well as Epiregulin and Betacellulin) can also bind and activate ErbB3 and/or ErbB4 (Arteaga & Engelman, 2014). Therefore, we cannot exclude the possibility that other ligands, for instance acting in a combinatorial fashion, could also have a role in Schwann cell‐mediated remyelination of central axons after SCI. Furthermore, a recent comparative transcriptome profiling analysis revealed that there is a complex interplay between the injury microenvironment and central progenitor cells that influences fate decisions (Ulanska‐Poutanen et al., 2018). Another complexity is added by recent findings suggesting significant heterogeneity among PDGFRα‐expressing progenitor cells, identifying a PDGFRα‐expressing population distinct from OPCs that resides along blood vessels (Marques et al., 2016). Given both the inherent heterogeneity (Marques et al., 2016) and adaptive heterogeneity (Ulanska‐Poutanen et al., 2018) of these cells, we cannot exclude the possibility that other processes after SCI may be altered due to ErbB receptor deletion on PDGFRα‐expressing progenitors, such as for instance injury‐induced angiogenesis (Marques et al., 2016; Russell, Stern, Polverini, & Bender, 1999). In relation to this, diverse roles for Nrg1 in mediating SCI pathology have also recently been highlighted, with evidence showing an astroglia‐ and immunomodulatory role of Nrg1 that supports neurological recovery after injury (Alizadeh et al., 2017; Alizadeh, Santhosh, Kataria, Gounni, & Karimi‐Abdolrezaee, 2018). However, regardless of the exact ligand signaling via ErbB3/4, and whether other pathophysiological parameters after SCI may also be affected, it is clear that ErbB receptor engagement is necessary for central progenitor cells of the PDGFRα lineage to transform into remyelinating Schwann cells in the injured spinal cord.

Our data suggest that most centrally occurring remyelinating Schwann cells that occupy a major part of the dorsal columns were derived from central progenitor cells of the PDGFRα lineage, with only a minor population remaining after preventing ErbB receptor signaling in these central progenitor cells. This minor population of Schwann cells seen in the dorsal columns after injury was most likely the preexisting peripheral Schwann cells that entered the injured spinal cord from the PNS via the dorsal roots. Previous fate‐mapping studies have demonstrated that neural crest‐derived peripheral Schwann cells that reside at the nerve roots infiltrate spinal cord lesions (Nagoshi et al., 2011). The notion of a major centrally derived de novo population of remyelinating Schwann cells and a smaller preexisting peripheral population of Schwann cells in the injured spinal cord is consistent with recent findings also using a fate‐mapping approach (Assinck, Duncan, Plemel, et al., 2017). Given that ~75%–80% of PDGFRα‐positive cells were recombined at 12 weeks post‐tamoxifen, it is likely that the Cre driver only removed ErbB receptors in ~75%–80% of the cells. Therefore, the remaining P0‐positive Schwann cell population present in the injured spinal cord after ErbB receptor ablation likely represents a mixed population of both infiltrating preexisting Schwann cells and newly formed Schwann cells derived from a central PDGFRα progeny where ErbB3/4 receptors remained intact, or potentially where ErbB was depleted but transformation still occurred. Importantly, the significant reduction in spontaneous locomotor recovery after injury upon preventing ErbB receptor‐dependent transformation of central progenitor cells into remyelinating Schwann cells, particularly in tasks for which dorsal column function is crucial (e.g., fine tuning of stepping ability, coordination, balance, and proprioception), indicates that dorsal column remyelination is crucial for fine tuning of restorative locomotor skills after SCI. Moreover, these data indicate that the minor migratory population of invading peripheral Schwann cells identified in the injured spinal cord in previous studies (Assinck, Duncan, Plemel, et al., 2017) plays a negligible role in this spontaneous myelin repair and functional recovery after SCI. The functional importance of Schwann cell‐mediated remyelination after SCI has recently been underlined by the finding that oligodendrocyte‐mediated remyelination following SCI is not required for spontaneous locomotor recovery (Duncan et al., 2018). This may be due to the more favorable and efficient time course of Schwann cell remyelination, as compared with the delayed onset of oligodendrocyte remyelination, after SCI since the time course of Schwann cell remyelination coincides with the major part of locomotor recovery (Duncan et al., 2018; James et al., 2011). Furthermore, peripheral type remyelination following transplantation of human olfactory mesenchymal stromal cells into a spinal cord lesion was shown to be associated with earlier recovery of interlimb co‐ordination (Lindsay et al., 2017).

The capacity of central progenitor cells to generate PNS‐like myelinating cells offers an interesting potential therapeutic avenue that remains poorly explored. We now provide direct mechanistic evidence that ErbB tyrosine kinase receptor signaling regulates phenotypic modulation of central progenitor cells. In the future, we anticipate that enhancing Nrg1‐ErbB tyrosine kinase signaling may accelerate and improve remyelination and may also allow identification or validation of key transcriptional changes in central progenitor cells. These could themselves be targeted in future experiments to enhance the phenotypic conversion of progenitor cells toward a Schwann cell fate with functional myelinating activity in vivo, in CNS pathologies where demyelination occurs. Enhancing functional remyelination, for example, in poorly myelinated lateral and ventral axonal tracts has not been achieved in this research field. This would represent, in combination with other treatment approaches, a significant advance with therapeutic potential for improving outcome after SCI and other CNS disorders with demyelinating pathology.
